# Noninvasive assessment and quantification of tumor vascularization using [18F]FDG-PET/CT and CE-CT in a tumor model with modifiable angiogenesis—an animal experimental prospective cohort study

**DOI:** 10.1186/s13550-019-0502-0

**Published:** 2019-06-21

**Authors:** Martin Mirus, Sergey V. Tokalov, Andrij Abramyuk, Jessica Heinold, Vincent Prochnow, Klaus Zöphel, Jörg Kotzerke, Nasreddin Abolmaali

**Affiliations:** 1 Biological and Molecular Imaging, OncoRay - National Center for Radiation Research in Oncology, Medical Faculty Carl Gustav Carus, TU Dresden, Fetscherstraße 74, 01307 Dresden, Germany; 2 Department of Anaesthesiology and Critical Care Medicine, University Hospital Carl Gustav Carus at the Technische Universität Dresden, Institution under Public Law of the Free State of Saxony, Fetscherstraße 74, 01307 Dresden, Germany; 3 Department of Neuroradiology, Medical Faculty and University Hospital Carl Gustav Carus, TU Dresden, Fetscherstraße 74, 01307 Dresden, Germany; 4 Municipal Hospital Dresden-Neustadt, Department of Neurology, Industriestraße 40, 01129 Dresden, Germany; 5 Clinic for Obstetrics and Gynaecology, Klinikum Chemnitz, Flemmingstraße 4, 09116 Chemnitz, Germany; 6 Department of Nuclear Medicine, University Hospital Carl Gustav Carus, Fetscherstraße 74, 01307 Dresden, Germany; 7 Department of Radiology, Municipal Hospital and Academic Teaching Hospital of the Technical University Dresden, Dresden-Friedrichstadt, Friedrichstraße 41, 01067 Dresden, Germany

**Keywords:** [18F]FDG-PET/CT, Glucose metabolism, Vascularization in tumors, Molecular imaging, Tumor microenvironment, Tumor perfusion

## Abstract

**Background:**

This study investigated the noninvasive assessment of tumor vascularization with clinical F-18-fluorodeoxyglucose positron emission tomography/computed tomography and contrast-enhanced computed tomography ([18F]FDG-PET/CT and CE-CT) in experimental human xenograft tumors with modifiable vascularization and compared results to histology. Tumor xenografts with modifiable vascularization were established in 71 athymic nude rats by subcutaneous transplantation of human non-small-cell lung cancer (NSCLC) cells. Four different groups were transplanted with two different tumor cell lines (either A549 or H1299) alone or tumors co-transplanted with rat glomerular endothelial (RGE) cells, the latter to increase vascularization. Tumors were assessed noninvasively by [18F]FDG PET/CT and contrast-enhanced CT (CE-CT) using clinical scanners. This was followed by histological examinations evaluating tumor vasculature (CD-31 and intravascular fluorescent beads).

**Results:**

In both tumor lines (A549 and H1299), co-transplantation of RGE cells resulted in faster growth rates [maximal tumor diameter of 20 mm after 22 (± 1.2) as compared to 45 (± 1.8) days, *p* < 0.001], higher microvessel density (MVD) determined histologically after CD-31 staining [171.4 (± 18.9) as compared to 110.8 (± 11) vessels per mm^2^, *p* = 0.002], and higher perfusion as indicated by the number of beads [1.3 (± 0.1) as compared to 1.1 (± 0.04) beads per field of view, *p* = 0.001]. In [18F]FDG-PET/CT, co-transplanted tumors revealed significantly higher standardized uptake values [SUVmax, 2.8 (± 0.2) as compared to 1.1 (± 0.1), *p* < 0.001] and larger metabolic active volumes [2.4 (± 0.2) as compared to 0.4 (± 0.2) cm^3^, *p* < 0.001] than non-co-transplanted tumors. There were significant correlations for vascularization parameters derived from histology and [18F]FDG PET/CT [beads and SUVmax, *r* = 0.353, *p* = 0.005; CD-31 and SUVmax, *r* = 0.294, *p* = 0.036] as well as between CE-CT and [18F]FDG PET/CT [contrast enhancement and SUVmax, *r* = 0.63, *p* < 0.001; vital CT tumor volume and metabolic PET tumor volume, *r* = 0.919, *p* < 0.001].

**Conclusions:**

In this study, a human xenograft tumor model with modifiable vascularization implementable for imaging, pharmacological, and radiation therapy studies was successfully established. Both [18F]FDG-PET/CT and CE-CT are capable to detect parameters closely connected to the degree of tumor vascularization, thus they can help to evaluate vascularization in tumors noninvasively. [18F]FDG-PET may be considered for characterization of tumors beyond pure glucose metabolism and have much greater contribution to diagnostics in oncology.

## Background

The fundamental importance of angiogenesis for tumor growth is well recognized for more than 40 years [1]. The critical size solid tumors may reach without neoangiogenesis is 1–2 mm^3^ [2, 3]. Growth beyond this volume cannot be supported exclusively by diffusion of oxygen and nutrients from the tumor environment but requires direct blood supply. By switching to an angiogenic phenotype, tumors start developing their own vessel system that allows further tumor progression. The result is an accelerated growth of the primary tumor, often accompanied by the spreading of tumor cells (metastases). Our understanding of the relationship between cancer, neovascularization, and metabolism is still limited. Nevertheless, characterization of tumor traits such as vascularity by imaging has been demonstrated to be prognostic for therapy response and can be utilized for rating prognosis [4–7]. That is why imaging of vascularization in tumors is so important. Reliable tumor models are a vital part of preclinical investigations and animal experiments are expected to be more relevant to the clinical situation than in vitro studies [8]. F-18-fluoro-2-deoxy-d-glucose ([18F]FDG) positron emission tomography (PET) visualizes the accumulation of a radioactive-labeled tracer in the body [9, 10]. In result, [18F]FDG radioactivity accumulates inside the hot spots of glucose metabolism, which can be detected by PET. The co-registration with computed tomography (CT) generates images of both high spatial resolution and morphological information.

In 1924, Otto Warburg described that cancer cells can be such a hot spot of glucose metabolism [9]. In normal cells, the influx of nutrients (e.g., glucose or glutamine) and the proliferation of the cells are regulated, among other things, by growth factors and interaction with extracellular matrix. Cancer cells reach a degree of independence from these external conditions [11]. The reason why cancer cells exhibit such a high demand for glucose is still focus of research [12, 13].

Several studies focused on imaging hypoxia in tumors as a possible reason for the high glucose demand. The most investigated PET tracer for hypoxia is fluoromisonidazole (FMISO). Newer tracers such as hydrophilic flortanidazole (HX4) were evolved to improve pharmacokinetics and imaging [14]. Due to their different profiles, it is hard to compare the different markers, what hamper conclusions drawn from hypoxia to glucose demand [14]. Targeting other structures involved in the glucose metabolism as glucose uptake transporters (GLUTs) or transcriptional factors (e.g., long-coding RNAs) are further examples for subjects of studies.

Due to their constitutively uptake of glucose, glutamine, and amino acids, cancer cells facilitate an uncontrolled proliferation while reducing the danger of lacking nutrients [11]. Proliferating cancer cells have a high demand for biosynthesis of lipids, proteins, and sugars. Many of these biosynthetic reactions need a source of reducing equivalents as NADPH. NADPH can be generated within the glycolysis. This makes it necessary to save carbon from reactions in the tricarboxylic acid cycle (TCA) and to increase glycolysis. By labelling carbon, it was possible to detect that glucose did not contribute much to the carbon used for generation of biomass but for ribose production for DNA and RNA [15]. Incorporated amino acids are the main contributors to the biomass [15]. Thus glucose is used to generate both precursor molecules for branching pathways and NADPH as reducing equivalent enabling other biosynthetic reactions [15]. By circumventing the mitochondrial tricarboxylic acid cycle (TCA) in favor of glycolysis cancer cells save metabolites which then can be used for generating DNA, membrane lipids, proteins, and carbohydrates synthesized from the glycolytic pathway [16]. Another consequence of increased glycolysis is the accumulation of lactate in the microenvironment of tumor cells. Research suggests that this support the tumor growth by attenuation of T cells and monocytes [15]. Furthermore, the lactate in the tumor surrounding promotes angiogenesis and may sustain tumor invasiveness.

Whatever the reason, visualizing this high consumption of glucose with [18F]FDG-PET became clinical routine. [18F]FDG-PET is still the most widely used radioactive tracer in oncological PET studies and helps to assess plenty of malignancies [17]. But questions remain: why do cancer cells exhibit this high demand for glucose and what do differences between tumors in the need for glucose mean? Might there be a connection between glucose requirements and other therapeutically relevant characteristics of the tumors? Maybe the glucose metabolism is connected to the degree of vascularization in a tumor? Imaging glucose metabolism means to image much more than pure glucose consumption [18, 19]. Consequently, the vascularization levels of tumors, how intravascular drugs could reach or affect the tumor, may be assessable. But the studies are still unclear and must face different challenges. The influence of both different histologies and different cell lines between the patients are some of these challenges.

The purpose of the present study was to investigate the impact of tumor vascularization on F-18-fluorodeoxyglucose positron emission tomography/computed tomography ([18F]FDG-PET/CT) imaging and contrast-enhanced computed tomography (CE-CT) imaging in human lung cancer xenograft tumors that provide different levels of vascularity within the same cell line. Standardized parameters from clinically established imaging techniques were correlated with histology and immunohistology.

## Methods

To explore the impact of vascularization on imaging modalities, a human xenograft tumor model consisting of one human cancer cell line but with alterable vascularization was used in this study. Due to the co-administration of endothelial cells and vascular growth promoters, the manipulation of the vascularization levels [20] of tumors arose from the same cell line was accomplished. In result, the specific influence of vascularization on imaging parameters could be investigated (growth rates, standardized uptake values (SUV), metabolic volumes, microvessel density (MVD), number of intravascular beads, CT volumes, CT vital tumor volumes, contrast enhancement in CT). In this manner, the challenge of other studies excluding influences of different cell lines as the size of cells or nuclei, gene expression, microenvironment, and other traits could be overcome in this study.

### Cells, animals, and tumor transplantation

Two human non-small-cell lung cancer (NSCLC) cell lines (A549 and H1299) were examined and rat glomerular endothelial (RGE) cells were used to manipulate vascularization levels. Cells were cultivated in high-glucose Dulbecco’s modified Eagle medium (DMEM) with heat-inactivated 10% fetal calf serum and 1% non-essential amino acids at 37 °C in a humidified atmosphere containing 7% CO_2_ as published before [21].

**Fig. 1:**
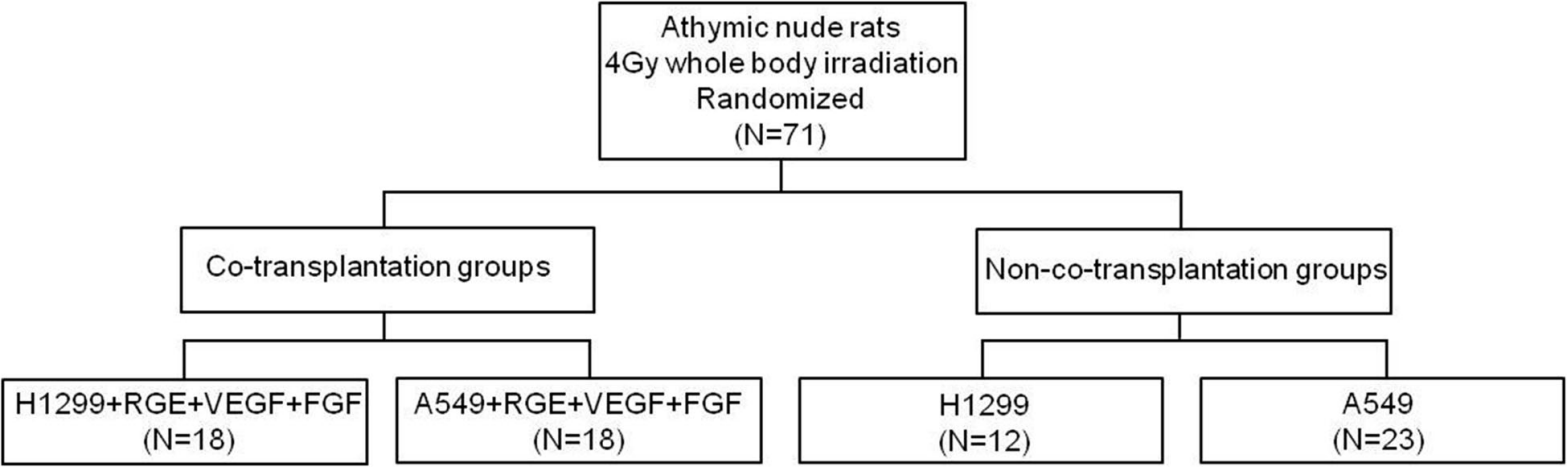
Experimental Groups. Survey of the four experimental groups with the different compounds of the subcutaneously injected cell suspensions. Two different tumor cell lines (H1299 and A549) and co-transplanted vascular growth promoters (*RGE* rat glomerular endothelial cells, *VEGF* vascular endothelial growth factor, *FGF* fibroblast growth factor)

Four- to six-week-old athymic nude rats were used. Animals received food and water ad libitum. To allow standardized growth conditions by decreasing residual immune response, all rats received a uniform whole body irradiation of 4 Gy [22, 23]. The rats in the animal experimental prospective cohort study were assigned randomly to one of four experimental groups (Fig. 1). The tumor cells were transplanted 48 h after irradiation by a subcutaneous injection of 200 μl tumor cell solution (5 × 10^6^ tumor cells) into the rat’s right lower limb. In both groups with modified vascularity, this tumor cell solution contained additionally 2 × 10^6^ RGE cells, 160 ng recombinant human vascular endothelial growth factor 165 (rHu VEGF-165), and 320 ng recombinant human fibroblast growth factor b (rHu FGF-b; Fig. 1).

After transplantation, the animals were weighted every other day, the transplantation region was inspected, and the tumor’s length (a) and width (b) were measured by caliper (volume = 1/6 ∙ π ∙ a ∙ b^2^). As soon as any tumor had reached a maximal size of 20 mm in one dimension, the rat was subjected to the multimodal imaging protocol, but not later than 49 days after transplantation.

The approval by the local animal care committee was obtained in accordance with the institutional guidelines and the German animal welfare regulations (24D-9168.11-1/2007-3).

### Imaging techniques

The imaging of tumors included [18F]FDG-PET/CT, CE-CT, and histological examinations.

**Fig. 2:**
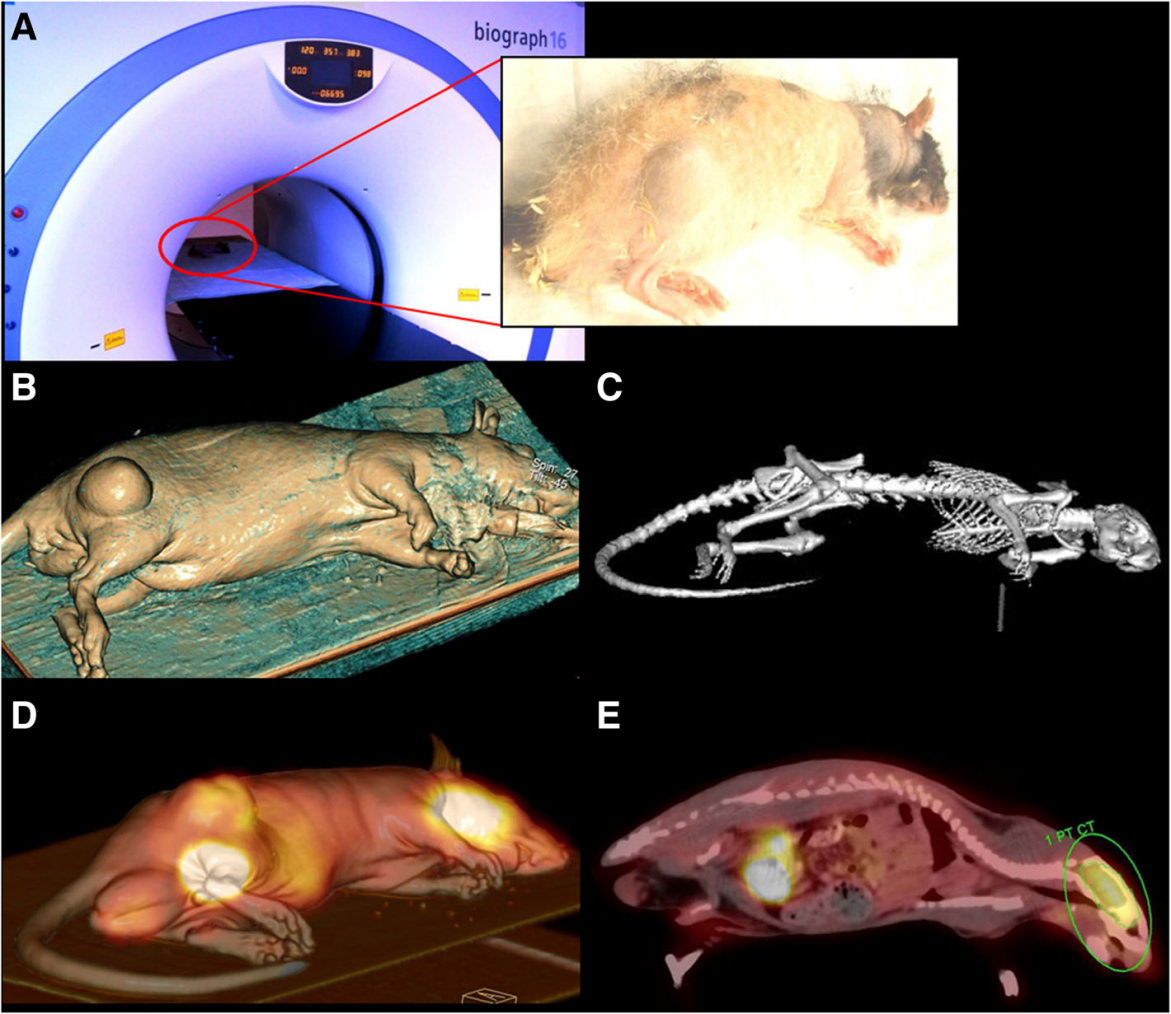
**a**–**e** Clinical scanners for preclinical research—[18F]FDG-PET/CT. **a** Tumor-bearing nude rat while scanning in PET/CT “biograph 16 Hi-Rez” (Siemens Healthineers). Narcotized rat carrying the tumor at the rat’s right lower limb. **b** Reconstruction using soft tissue windowing by CT data. High image quality visualizes even surface details using clinical scanners, e.g., the tumor at the rat’s right lower limb. **c** Reconstruction using bone windowing by CT data. **d** 3d rigid Fusion of [18F]FDG-PET and CT data. Position as in **a**–**c** with co-registration of [18F]FDG-PET. High [18F]FDG activity in four locations, highest at brain and urinary bladder. Less intensity can be seen in the abdominal part, near to the primary [18F]FDG injection and at the tumor site at the right lower limb. **e** Sagittal view of [18F]FDG-PET/CT scan. Green region of interest to analyze [18F]FDG-uptake in the brain

For [18F]FDG-PET/CT and CE-CT the PET/CT, “biograph 16 Hi-Rez” (Siemens, Knoxville, TE, USA; Fig. 2a) and “syngo TrueD” software were used. After 24 h of fasting, the rats received an intraperitoneal (i.p.) injection of 11.1 MBq (SD 1.2 MBq) [18F]FDG followed by intraperitoneal injection of ketamine (90 mg/kg) and xylazine (10 mg/kg) 20 min later for anesthesia [24]. In this study, the i.p. way was used due to the very easy and safe injection compared to intravenous (i.v.) injection into the tail vein of the rats. Due to the equal blood concentration of [18F]FDG reached by the i.p. injection [25, 26], the authors neither expect a better nor a worse visualization of tumors in respect to injection technique. Subsequently, a catheter was implanted to the nude rat’s jugular vein (24 gauge peripheral venous catheter, B. Braun Melsungen AG, Melsungen, Germany). The catheter was used for i.v. application of contrast agent (CM) Ultravist 370, Bayer Schering Pharma AG (Bayer AG, Leverkusen, Germany) during CT imaging protocols. Accomplished CT protocols were attenuation correction CT, plain CT without CM, and venous CT with CM. After the plain CT scan, the injection of CM started and 1 ml of CM was applied in 30 s. CT scanning started 20 s after the injection start. All CT protocols were carried with the animal in the same position.

The following scan protocols and specifications were used. PET: scan start 30 min post intraperitoneal [18F]FDG injection; matrix 256 × 256; voxel size 2.05 × 2.05 × 1.5 mm; slices 109; CT for transmission correction: 120 kV; 100 mAs; matrix 512 × 512; voxel size 0.47 × 0.47 × 0.75 mm; CT: 80 kV, 100 mAs, matrix with 512 × 512 pixel, voxel resolution of 0.42 × 0.42 × 0.75 mm.

### Histology and immunohistology

After PET and CT imaging, 200 μl of 2.4 x 10exp7 fluorescent beads (diameter 2.5 μm, excitation wave length 633 nm; G. Kisker GbR, Steinfurt, Germany) were injected via the jugular vein of the rats. The rats were sacrificed 2 min after injection. Subsequently, the organs and the tumor were removed and conserved in liquid nitrogen. Representative slices (thickness 10 μm) of organs were stained with hematoxylin-eosin (HE). Tumor slices were additionally stained with cluster of differentiation 31 (CD-31) antibody to quantify microvessel density (MVD).

### Image analysis

Histology and bead counts (number of beads per field of view) served as standard of reference. The beads were examined at fivefold magnification using a fluorescent microscope in 20 fields of view (FOV). For CD-31 staining, five hot spots for each tumor were digitally recorded at 20-fold magnification and the number of CD-31 positively stained structures was evaluated as published before [27], with MVD estimated as the number of stained structures per mm^2^. The estimated parameters were beads/FOV and MVD/mm^2^.

**Fig. 3:**
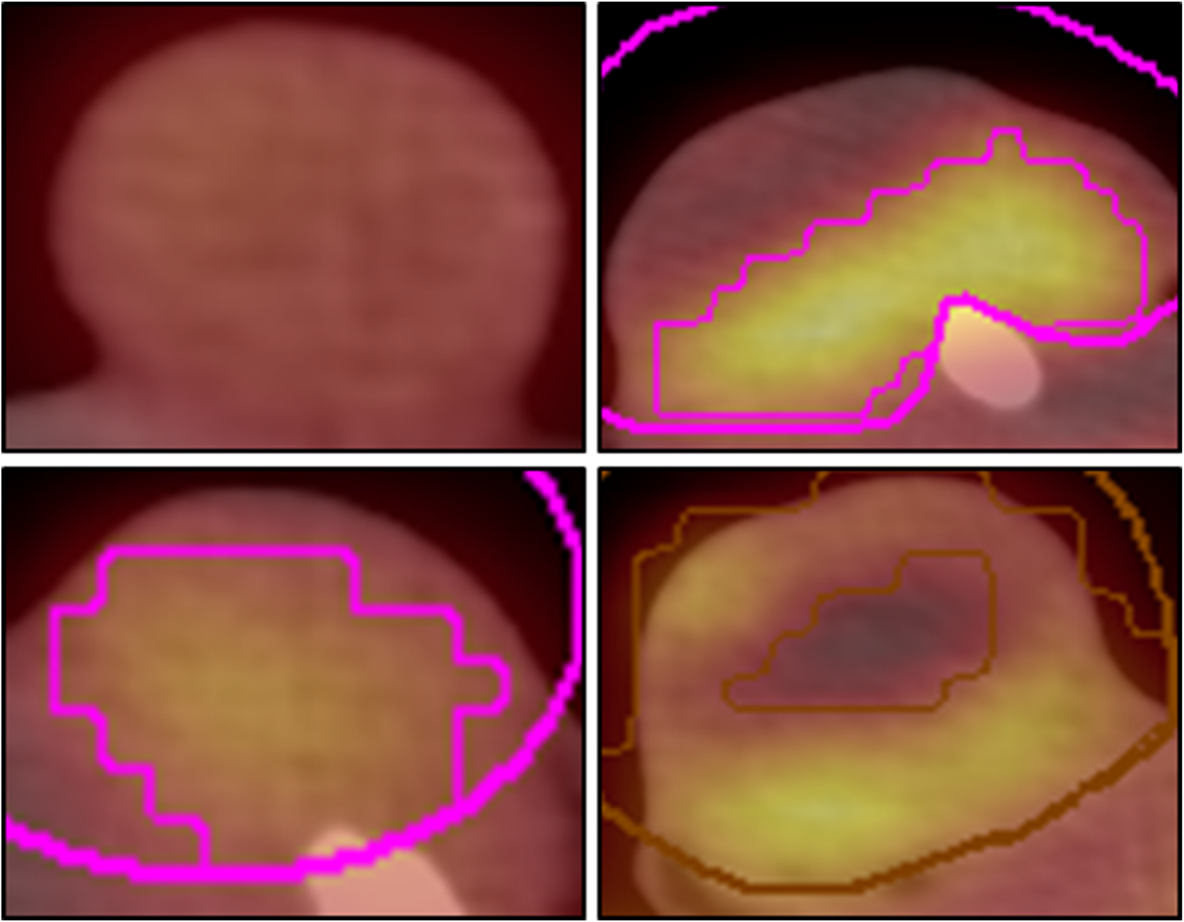
Imaging tumors with [18F]FDG-PET/CT. The upper and lower rows depict images from cell line A549 and H1299, respectively; the left and right columns depict images for non-co-transplanted and co-transplanted tumors, respectively. Left column: lower activity with homogenous pattern of [18F]FDG-activity in non-co-transplanted tumors; right column: in contrast higher activity with heterogeneous pattern of [18F]FDG-activity in co-transplanted tumors

For analysis of [18F]FDG-PET/CT data, tumors were visually assigned to one of two groups: heterogeneous or homogenous pattern of [18F]FDG distribution (Fig. 3). Contours were drawn, and SUVs were estimated for both the brain (Fig. 2e) and a representative part of the muscles in the left lower thigh. Contours were drawn surrounding the tumor in the CT images and the SUV in the tumor was estimated for three different lower limit values—A: 50% of brain SUVmean, B: 50% of tumor SUVmaximum, and C: 1.5 x muscle SUVmean [28, 29]. By applying each lower threshold (A, B, C), three corresponding metabolic active volumes were estimated (tumor volume exhibiting a SUV of the threshold or higher). Relative active volume was estimated by relation of the active volume to the gross tumor volume in CE-CT. In CE-CT series, an outline was drawn around the contrast-enhanced tumor in all slices, including outer edge of tumor, skin, and CM-lacunas but excluding bones and vessels without contact to the tumor. Limits for analysis of the entire tumor were − 50 ≤ Hounsfield Unit (HU) ≤ 350. By modifying these limits to + 50 ≤ HU ≤ 350 within the same outline, the characteristics of the non-necrotic (vital) parts of the tumors were investigated. Characteristics of the non-contrast-enhanced tumor were determined by copying this outline to the plain CT series.

Estimated parameters in [18F]FDG-PET/CT: minimum, maximum, and mean SUV; metabolic active volume; relative metabolic active volume; [18F]FDG-pattern; in CE-CT: tumor volume, vital and relative vital tumor volume, contrast enhancement (CE) in vital tumor volume in relation to maximum HU in plain CT series.

### Statistical analysis

All statistical analyses were carried out with PASW 18 (Predictive Analytics SoftWare, IBM, Armonk, NY, USA) by comparisons between co-transplanted and non-co-transplanted tumors with and without regard of the tumor cell line. The Kolmogorov-Smirnov test was used to test for normality. The Mann-Whitney *U* test was used to analyze parameters that violated normality, whereas Student’s *t* test was used when normality was given. Spearman’s rank correlation coefficients were calculated to evaluate correlations. The Chi^2^-test was used to compare the results of [18F]FDG distribution pattern. A *p* value lower than 0.05 was considered to indicate a statistically significant difference.

## Results

### Cells, animals, and tumor transplantation

**Fig. 4:**
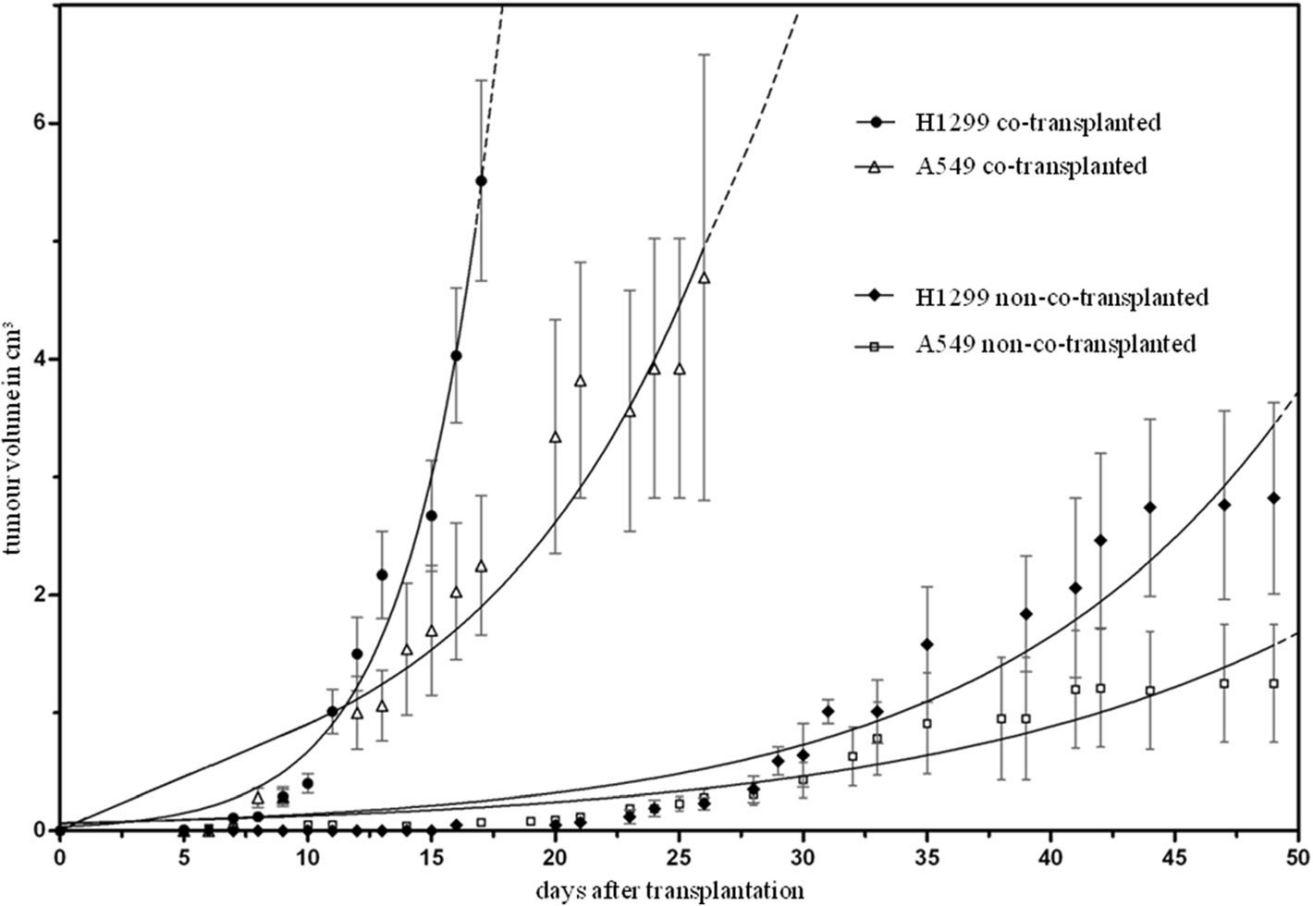
Growth of xenograft tumors. Increase of the xenograft tumor volumes of the non-small-cell lung cancer (NSCLC) cell lines A549 and H1299, estimated by in-vivo caliper measurements. In co-transplanted tumor groups vascular growth promoters (RGE cells, rHu-VEGF-165, rHu-FGF-b) were added to the transplanted tumor cells

**Fig. 5:**
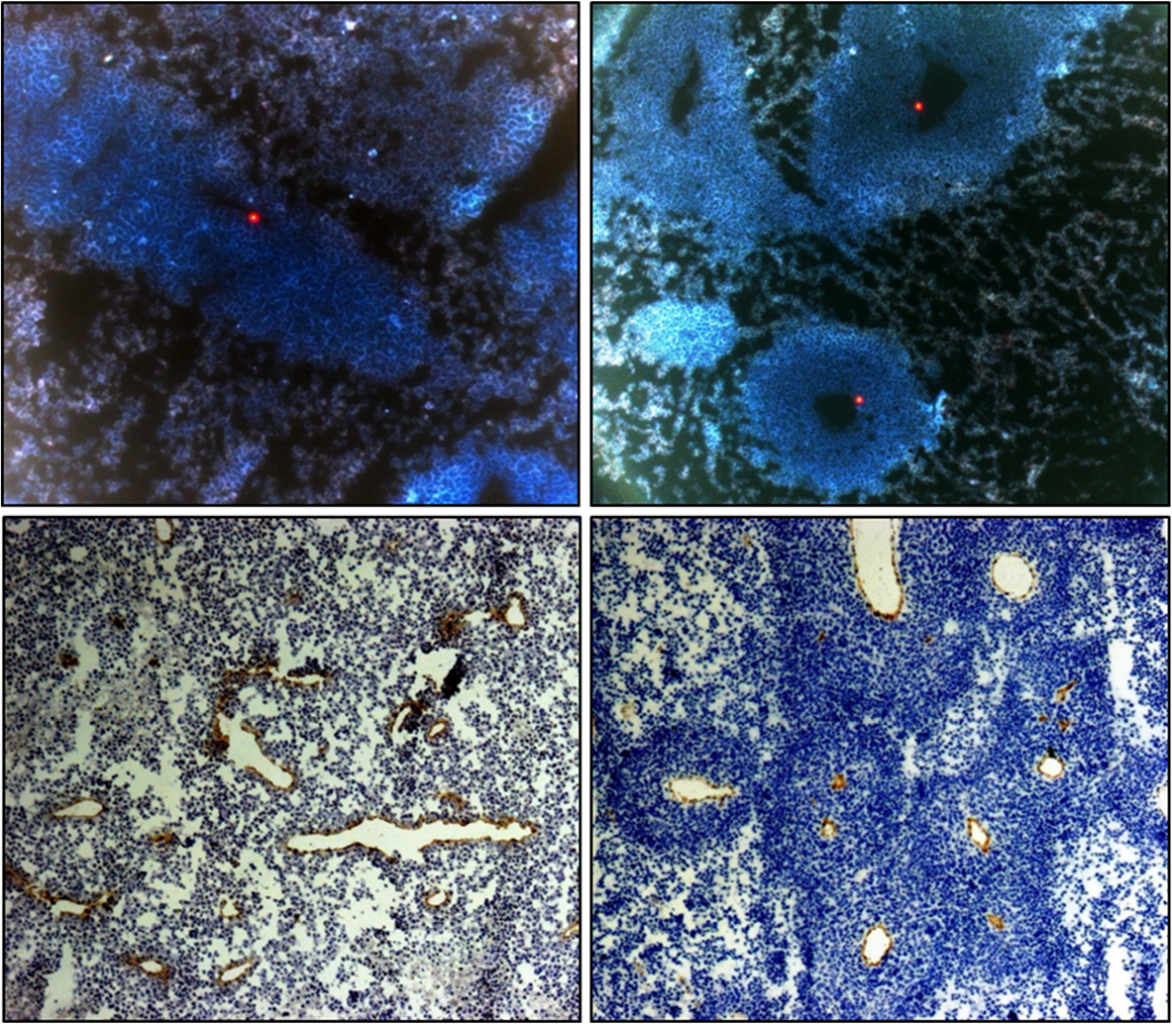
Histology and immunohistology. The upper and lower rows depict images from fluorescent microscopy and light microscopy with CD-31vessel marker, respectively. The left and right columns depict images of non-co-transplanted and co-transplanted tumors, respectively. In fluorescent histology, one red fluorescent bead is detectable within a part of compact tumor tissue in a non-co-transplanted tumor. In contrast, two fluorescent beads within clear visible vessel lumens in co-transplanted tumor. In light microscopy, the CD-31-positive areas (vasculature) stained in brown, and the gray/blue tumor stromal tissue is visible. In the non-co-transplanted tumor less compact tumor tissue developed and in contrast, brown vessel walls surrounded by blue compact vital tumor tissue in co-transplanted tumor

The co-transplanted tumors revealed a significantly faster growth compared to the non-co-transplanted tumors. This holds true for both tumor cell lines H1299 and A549 (Table 1; Fig. 4). Both the MVD (Table 1; Fig. 5) and the number of beads (Table 1; Fig. 5) were higher in co-transplanted tumors, while the number of beads in the investigated organs did not differ between the groups. Histology revealed that the central regions of the co-transplanted tumors often showed clusters of necrotic regions next to vessels with larger diameters (Fig. 5). Notably, the latter were often closely connected to clouds of CD-31-positive small vessels (Fig. 5). Both necrotic areas and CD-31-positive vessels with large diameter were rarely found in the center of non-co-transplanted tumors (Fig. 5).

**Table 1:** Growth and histology

		H1299			A549			All tumors		
		Non-co-trans-plantation	Co-trans-plantation	*p* value	Non-co-trans-plantation	Co-trans-plantation	*p* value	Non-co-trans-plantation	Co-trans-plantation	*p* value
In vivo										
Growth duration	Days	49.00 (20)	18.0 (11)	< 0.001	45.0 (10)	23.5 (15)	< 0.001	45.9 (7)	22.0 (12)	< 0.001
In vitro										
Beads	Number/FOV	1.10 (0.28)	1.3 (0.17)	0.009	1.1 (0.22)	1.3 (0.23)	0.032	1.1 (0.25)	1.3 (0.22)	0.001
MVD	Number/mm^2^	89.7 (65.49)	203.1 (185.49)	0.036	114.6 (81.98)	153.4 (112.75)	0.018	101.8 (63.3)	171.4 (120)	0.002

*FOV* field of view, *MVD* microvessel density

Tumor growth duration till endpoint and in vitro data derived from histology and immunohistology. Measurements were done as soon the tumors had reached a maximum diameter of 20 mm or after 48 days of growth, depending on which criterion was reached first. First and second column show results separated for both cell lines, third column show results for tumor cell lines merged together. Median (interquartile range)

### [18F]FDG-PET/CT

#### Pattern of accumulation of [18F]FDG-PET activity

PET/CT visualized that co-transplanted tumors (Fig. 3) exhibited a heterogeneous distribution pattern of [18F]FDG-activity more frequently as compared to the non-co-transplanted tumors (Fig. 3).

#### SUV in [18F]FDG-PET

Higher maximal, mean, and minimal SUVs in the co-transplanted as compared to the non-co-transplanted tumors were found in [18F]FDG-PET (Table 2, Fig. 3). Calculations were done with SUV threshold A (50% of brain SUVmean).

**Table 2:** Study results [18F]FDG-PET and CE-CT

		H1299			A549			All tumors		
		Non-co-trans-plantation	Co-trans-plantation	*p* value	Non-co-trans-plantation	Co-trans-plantation	*p* value	Non-co-trans-plantation	Co-trans-plantation	*p* value
CE-CT										
Tumor volume	cm^3^	1.94 (4.06)	6.98 (7.07)		0.17 (0.25)	6.95 (5.94)		0.28 (2.08)	6.98 (5.87)	
Vital volume	cm^3^	1.57 (3.10)	5.03 (4.73)		0.15 (0.22)	5.39 (3.93)		0.25 (1.60)	5.3 (4.62)	
Relative vital volume	%	86.61 (13.15)	74.53 (7.57)	0.036	85.71 (6.93)	80.65 (11.35)	0.008	85.71 (8.36)	77.73 (13.84)	< 0.001
CE in whole tumor	%	196.76 (55.13)	269.92 (55.61)	0.004	204.83 (45.80)	270.20 (56.03)	< 0.001	201.48 (46.31)	270.20 (50.20)	< 0.001
CE in vital tumor	%	215.42 (87.58)	366.87 (65.43)	< 0.001	217.38 (45.60)	336.54 (69.97)	< 0.001	217.07 (56.05)	340.55 (78.53)	< 0.001
Mean HU in vital tumor	HU	104.50 (25)	125.50 (19)	0.003	112.50 (15)	116.50 (12)	0.581	110.50 (16)	119.50 (15)	0.001
^18^F-FDG-PET										
Number of rats	*N*	10	18		21	18		31	36	
SUV_max_		1.19 (1.41)	2.43 (0.71)	0.003	1.04 (0.60)	3.16 (1.23)	< 0.001	1.14 (0.82)	2.76 (1.01)	< 0.001
SUV_mean_		1.16 (0.84)	1.80 (0.36)	0.011	1.04 (0.60)	2.04 (0.82)	0.001	1.11 (0.70)	1.89 (0.57)	< 0.001
SUV_min_		1.13 (0.58)	1.41 (0.41)	0.093	1.04 (0.54)	1.45 (0.58)	0.035	1.05 (0.56)	1.44 (0.47)	0.003
Metabolic active volume (thresh.A)	cm^3^	0.18 (0.95)	1.54 (3.92)	0.002	0.01 (0.00)	2.66 (3.75)	< 0.001	0.01 (0.20)	2.06 (3.70)	< 0.001
Relative met. Act. volume (thresh.A)	%	13.39 (19.54)	26.75 (21.62)	0.188	6.25 (6.37)	38.83 (31.81)	< 0.001	7.14 (11.86)	29.72 (30.30)	< 0.001
Metabolic active volume (thresh.B)	cm^3^	1.47 (2.06)	2.54 (1.14)	0.004	0.32 (0.20)	2.29 (2.19)	0.001	0.38 (1.33)	2.42 (1.32)	< 0.001
Relative met. Act. volume (thresh.B)	%	95.29 (120.29)	35.13 (16.62)	0.001	128.57 (109.85)	31.52 (14.15)	< 0.001	126.32 (115.56)	33.83 (15.01)	< 0.001
Metabolic active volume (thresh.C)	cm^3^	2.14 (3.51)	6.84 (4.31)	< 0.001	0.1 (0.37)	6.69 (5.28)	< 0.001	0.26 (2.60)	6.79 (4.28)	< 0.001
Relative met. Act. volume (thresh.C)	%	93.64 (81.19)	81.83 (23.58)	0.833	51.35 (90.29)	87.43 (22.91)	0.111	62.5 (90.06)	86.23 (22.66)	0.196

*HU* Hounsfield Unit, *CE*-*CT* contrast-enhanced computed tomography, [*18F*]*FDG*-*PET* F-18-fluorodeoxyglucose positron emission tomography, *threshold A* 50% of brain SUVmean, *threshold B* 50% of tumor SUVmaximum, *threshold C* 1.5 x muscle SUVmean

Study results in overview. First and second column show results separated for both cell lines, third column show results for tumor cell lines merged together. Median (interquartile range)

#### Metabolic active and relative metabolic active volume in [18F]FDG-PET/CT

The co-transplanted tumors revealed both significantly larger metabolic active volumes and relative metabolic active volumes as compared to the non-co-transplanted tumors (Table 2); calculations were done with SUV threshold A (50% of brain SUVmean).

### CT

#### Plain and contrast-enhanced imaging

**Fig. 6:**
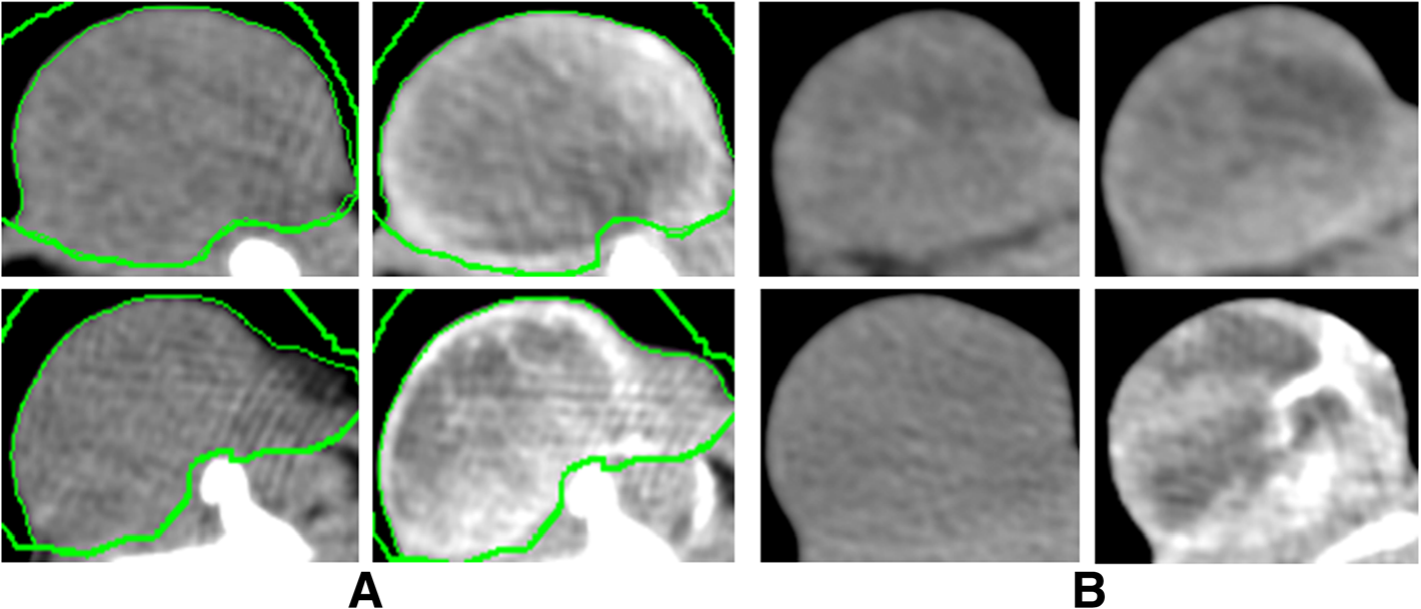
Imaging tumors with CE-CT and native CT. Panel **a** and **b** show details of xenograft tumors for cell line A549 and H1299, respectively. The upper and lower rows depict images for non-co-transplanted and co-transplanted tumors, respectively. The left and right columns in **a** and **b** depict images from native and contrast-enhanced images. Compare the different degree of contrast enhancement between non-co-transplanted (upper row) and co-transplanted (lower row) tumors. **a** Upper row: non-co-transplanted tumor with moderate contrast enhancement from native (left column) to contrast-enhanced (right column) image. Lower row: co-transplanted tumor with high contrast enhancement from native (left column) to contrast-enhanced (right column) image. **b** Upper row: non-co-transplanted tumor with modest contrast enhancement from native (left column) to contrast-enhanced (right column) image. Lower row: co-transplanted tumor with high contrast enhancement from native (left column) to contrast-enhanced (right column) image and vessel within the tumor becoming visible in co-transplanted contrast-enhanced image

**Fig. 7:**
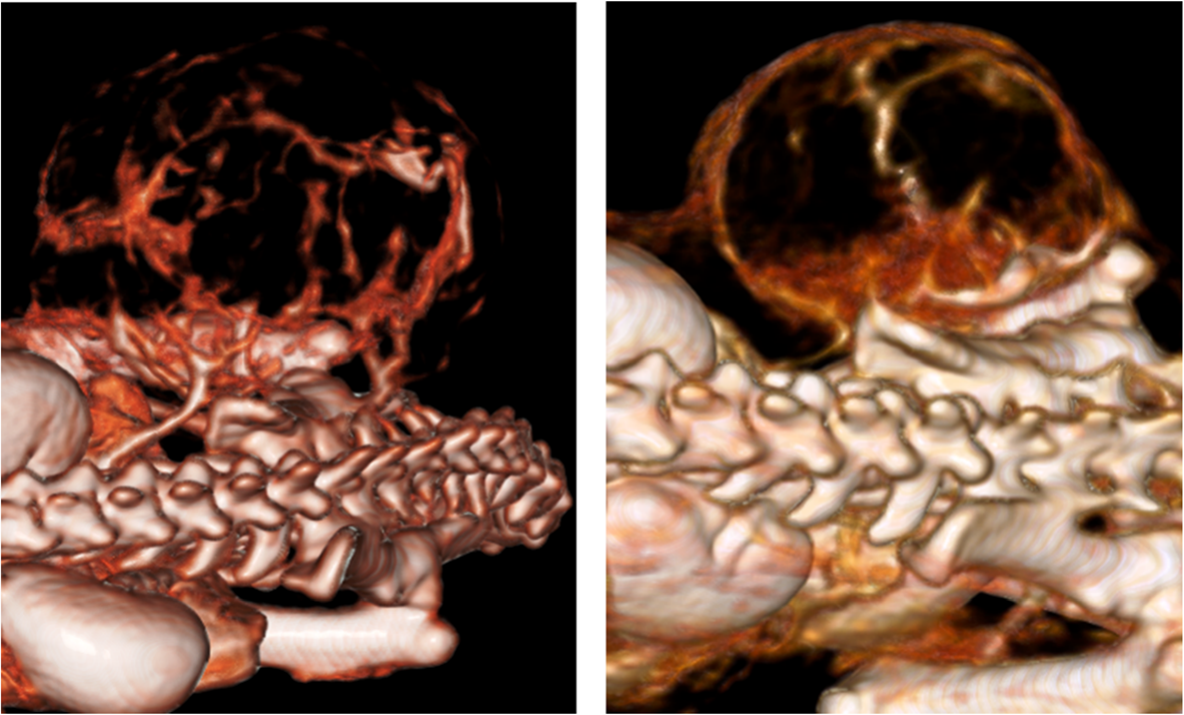
3d-reconstruction of CT-data. Reconstruction of contrast-enhanced data of two tumors of the co-transplantation group. A vessel originating from the paravertebral region (iliac artery) and supplying the tumor region and vessels within the tumors are clearly depicted

On plain CT scans, the co-transplanted tumors exhibited a trend toward lower HU values (30.5 ± 0.5 HU, mean ± standard deviation), whereas non-co-transplanted tumors had higher HU values (32 ± 1 HU). After injection of CM, the co-transplanted tumors revealed a higher contrast enhancement (120 ± 2.2 HU) than the non-co-transplanted tumors (111 ± 2 HU). This difference was significant (*p* = 0.001, Fig. 6, Table 2). Reconstruction of this contrast-enhanced imaging data enables qualitative visualization of vessels within tumors (Fig. 7).

#### Relative vital tumor volume and contrast enhancement in vital tumor in CE-CT

The co-transplanted tumors revealed smaller relative vital tumor volumes as compared to the non-co-transplanted tumors. However, the contrast enhancement in the vital tumor parts in the co-transplanted tumors was significantly higher than in vital tumor parts in the non-co-transplanted tumors (Table 2, Fig. 6).

### Correlation analyses

**Fig. 8:**
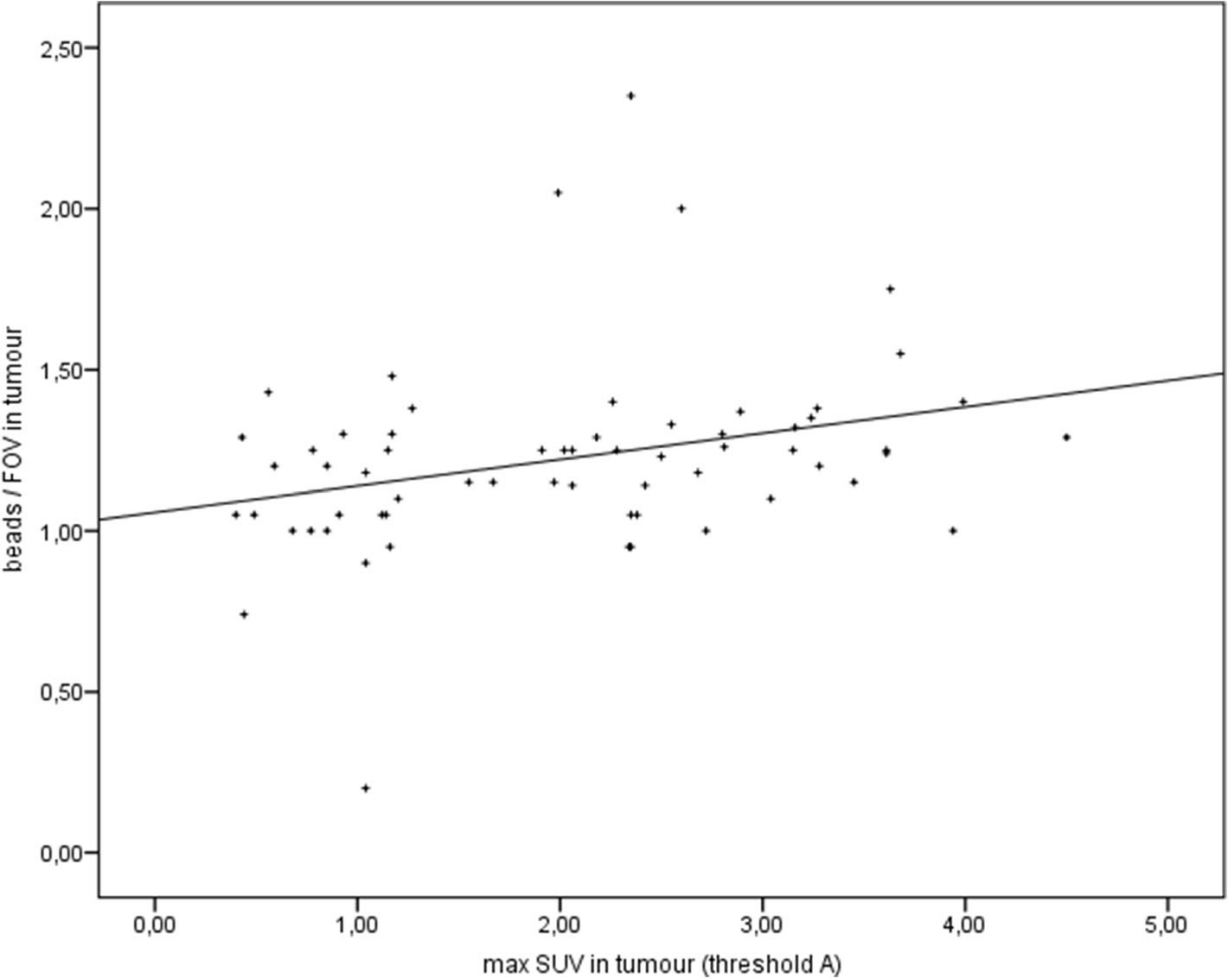
Correlation. Spearman’s rank correlation between the maximal SUV in [18F]FDG-PET and the fluorescent beads (histology, [beads/FOV]); *r* = 0.353, *p* = 0.005)

**Fig. 9:**
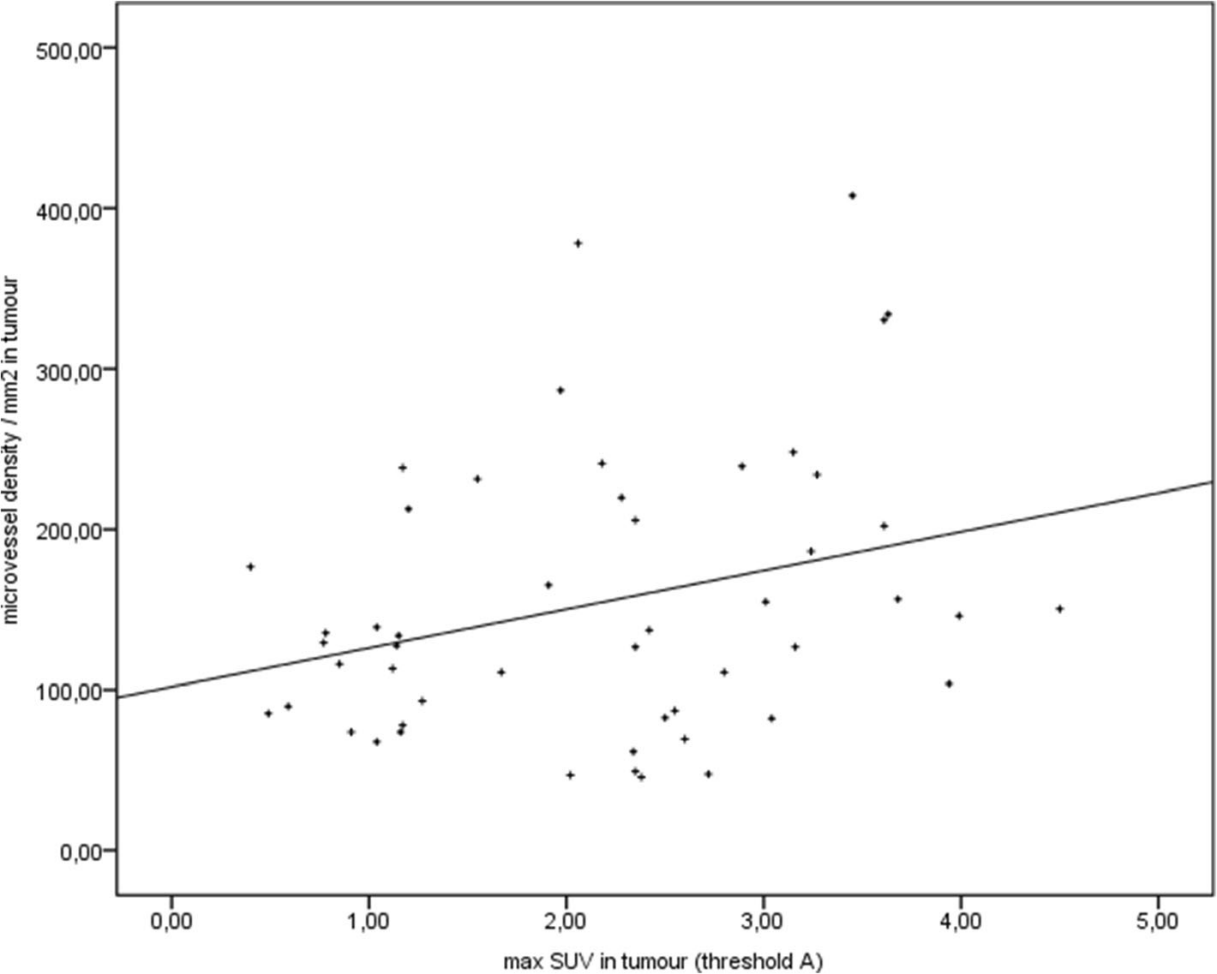
Correlation. Spearman’s rank correlation between the maximal SUV in [18F]FDG-PET and the microvessel density (MVD, [MVD/mm^2^]); *r* = 0.294, *p* = 0.036

**Fig. 10:**
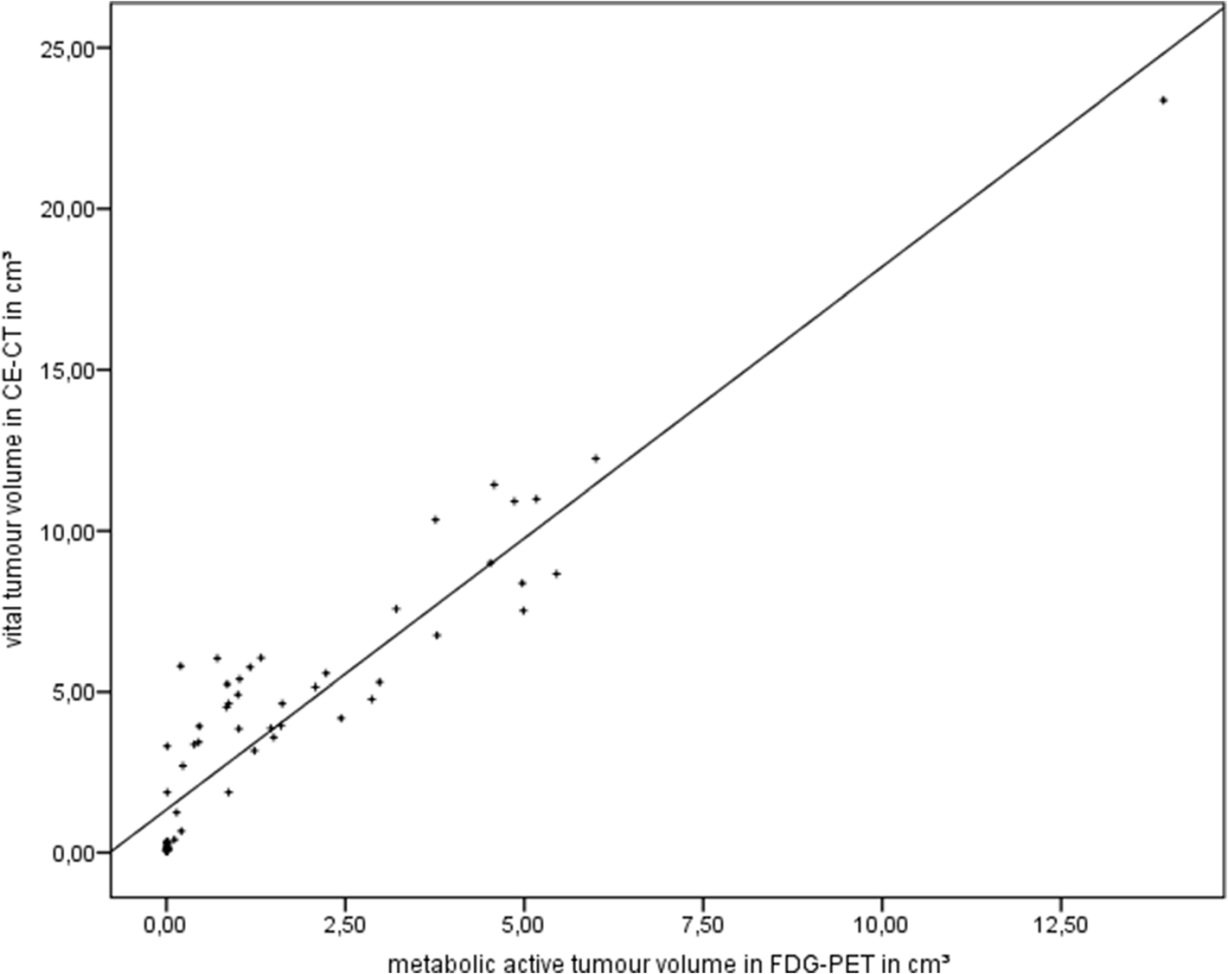
Correlation. Spearman’s rank correlation between the metabolic active tumor volume in [18F]FDG-PET and the vital tumor volume in CE-CT; *r* = 0.919, *p* = 0.000

**Fig. 11:**
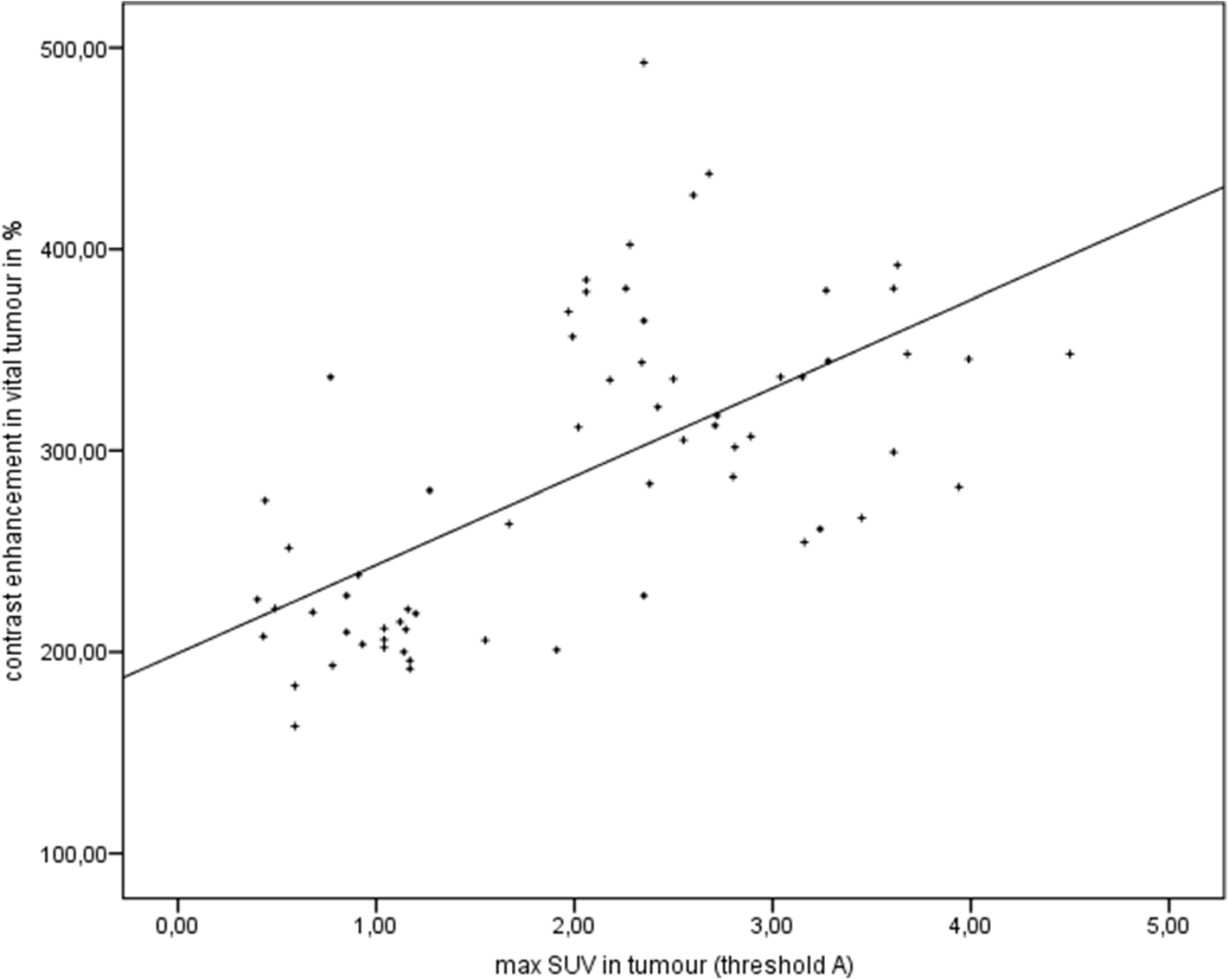
Correlation. Spearman’s rank correlation between the maximal SUV in [18F]FDG-PET and the contrast enhancement in vital tumor in CE-CT; *r* = 0.635, *p* = 0.000

There were significant correlations between [18F]FDG-PET/CT, CE-CT, and histology. The correlation between the maximal SUV in tumors and the beads/FOV as assessed by fluorescent microscopy was found to be significant in all tumors (*p* = 0.005, *r* = 0.353, Fig. 8). Results are similar for the correlation between the maximal SUV and the microvessel density as assessed by CD-31 staining in immunohistology (*p* = 0.036, *r* = 0.294, Fig. 9). The correlation between both histological quantifications of vascularization (beads and CD-31) also was significant (*p* = 0.007, *r* = 0.376). The correlation between the metabolic active volumes in [18F]FDG-PET and the vital volumes in CE-CT was found to be significant in all tumors too (*p* < 0.001; *r* = 0.919, Fig. 10); calculation was done with SUV threshold A (50% of brain SUVmean). Furthermore, the correlation between the maximal SUV in tumors and the contrast enhancement in the vital part of the tumors was found to be significant in all tumors (*p* < 0.001; *r* = 0.635; Fig. 11).

## Discussion

A purpose of this study was to expand the information obtained from [18F]FDG-PET/CT beyond glucose metabolism. The authors were driven by the idea that besides the investigation of new imaging tracers and methods, the improvement of established ones is of tremendous importance. [18F]FDG-PET/CT is such an established clinical method, imaging glucose uptake. Glucose uptake and metabolism are regulated by a lot of molecular pathways, which also affect angiogenesis in cancer [12, 30–32]. This provides a basis for a closer look for the molecular links between glucose and vascularization. This study aimed to provide an insight how both angiogenesis and vascularization impact glucose uptake and metabolism in tumors. The authors wanted to explore the connection between glucose imaging and vascularization parameters, in order to understand which conclusions can be drawn from clinical established [18]FDG-PET/CT concerning tumors vasculature.

### Glucose uptake and its influencers

Glucose and [18F]FDG accumulation in tumors dependent, among other things, on tumor vasculature (i.e., deliver the tracer to the region of interest), on glucose transporters (i.e., intracellular uptake of the tracer), on phosphorylation (i.e., trapping the trace into the cell), and on metabolism (i.e., need and use of the tracer) [33]. The amplified expression of various transmembrane glucose transporters (GLUTs) and intracellular enzymes (e.g., hexokinases, HK) are common features in a multitude of tumors. This seems necessary because of higher glucose consumption due to altered metabolism (aerobic glycolysis) in tumors [9, 34]. Both GLUTs (esp. GLUT-1, GLUT-3) and the high active hexokinases (esp. HK-II) are two reasons for the increased influx and trapping of [18F]FDG in tumor cells [35–38]. All of this influences the uptake of [18F]FDG and thus the measured imaging parameters. This must be taken into consideration when interpreting [18F]FDG-PET/CT data for additional conclusions about vascularization.

### Other approaches to image vascularization

A more focused approach assessing vasculature is the application of an intravascular tracer not being influenced by transporter proteins or metabolism. Such tracers are delivered with the blood in the vessels. This study used intravenous fluorescent beads as such robust tracer, with the need of post-mortem quantification. Linking these tracers to radioactive markers enables in vivo analysis. The preparation of such a tracer (e.g., [99mTc]Phytate, starch-based microparticles, [99mTc]Albumin) is rather complex and the behavior of these tracers in the body have to be learned in clinical studies [39–41]. Of course, these methods are less influenced by confounders compared to above mentioned [18F]FDG analysis, but in return they can hardly provide any further information as [18F]FDG-PET/CT does. For example, they cannot image the activation of metabolic pathways which are connected to angiogenesis. Due to the molecular link between glucose uptake and angiogenesis, [18F]FDG-PET may do.

Another approach to observe angiogenesis is to identify hypoxic regions driven by the idea that hypoxia causes both angiogenesis and high glucose metabolism (anaerobic glycolysis) in tumors. The transcriptional activator hypoxia-inducible factor-1 (HIF-1) normally becomes stabilized in cells under hypoxic conditions, and then leads to angiogenesis. However, in cancer, some data do not show correlations between the expression of hypoxia-inducible factor (HIF-1) and regions defined as hypoxic [30, 42]. Imaging hypoxia then barely helps to capture angiogenesis. But what if connection between metabolism and angiogenesis is stronger than between hypoxia and angiogenesis? Finding hypoxic regions would then be less important [30]. Detecting glucose metabolism using [18F]FDG-PET/CT might then indicate for angiogenesis. This may refer to a closer connection between angiogenesis and glucose metabolism. In this study, the authors decided to explore this connection. They choose [18F]FDG as imaging tracer to investigate whether molecular links between glucose and vascularization are reflected in parameters derived in [18F]FDG-PET.

### Briefly: molecular links between glucose and vessels

The molecular links between the pathways of angiogenesis and glucose uptake and metabolism provide the foundation for this study. Therefore, in the following, it is worth putting the spotlight briefly on the molecular level.

Mutated pathways in cancers often involve both metabolism and angiogenesis; the transcription factors HIF-1 and p53 are two important examples. Due to mutation, the overexpression of HIF-1 has been found in various cancers [12, 34, 43]. It leads to activation of glycolysis and to inhibition of oxidative phosphorylation, thus HIF-1 influences glucose metabolism [12, 34]. HIF-1 also drives the synthesis of VEGF, which promotes angiogenesis [34]. The mutation of the P53-gene in cancer cells lead to amplified expression of GLUT-1, GLUT-3, and GLUT-4 and consequently to increased glucose uptake in cancer cells and in consequence to a higher [18F]FDG uptake in PET [36, 37, 44]. In addition, the deficiency of p53 leads to the upregulation of proangiogenic and to the downregulation of antiangiogenic factors [45]. Thus the (functional) inactivation of p53 also influences both metabolism and angiogenesis [16, 34, 44–46].

This exemplary reflects the close relation between angiogenesis and glucose (metabolism). So, [18F]FDG-PET should detect much more than just the metabolic activity or accumulation of tracers only due to perfusion. The present study demonstrated a significant influence of tumor vascularization on estimated parameters in [18F]FDG-PET.

### The angiogenic switch: start of new vascularization and its manipulation in this study

An important condition for both fast and continuous tumor growth is the angiogenic switch. The tumor model used in this study increased the probability of a more intense angiogenic switch leading to a better vascularization in these tumors. The histological and immunohistological results support this interpretation, the co-transplanted tumors revealed the better vascularization (higher MVD and higher number of beads). Tumor vessels are not as functional as normal vessels, but they are perfused [47]. This is proven by the higher number of fluorescent beads in tumors with higher MVD, as beads can reach the tumor only by perfusion. Thus, co-transplantation of RGE cells and growth promoters lead to improved vascularization of the tumors. Because the cell line and the amount of tumor cells remained the same, the faster growth rates in the co-transplanted tumors can be explained by a better supply with oxygen and nutrients due to better vascularization. Some tumors in the non-co-transplantation group also reached comparable sizes; however, the number of tumors that did and their rate of growth were much smaller. This indicates an occurred but less intense angiogenic switch in these tumors.

### Connection of [18F]FDG-PET, CE-CT, and vascularization

In this study, [18F]FDG-PET and CE-CT parameters seem to be connected to tumor vascularization. Different explanations must be taken into consideration.

#### First explanation

The better blood supply in tumors leads to faster growth in some tumor areas. Due to their fast growth rate but their confused and unorganized growth of vessels [47], the high vascularized tumors developed areas of critical shortage with oxygen and nutrients. This limited supply led to necroses in some parts of the larger tumors. Parts that initially were supplied by diffusion of oxygen, but with the growth of the tumor diffusion became insufficient and cells got necrotic. Additionally, new evolving vessels could grow into these necrotic parts and generate a similar pattern. In the well-vascularized large tumors, high diameter vessels were found leading through regions without any further vessels. Thus, oxygen supply and nutrition in these regions was limited to diffusion from one big single vessel. This caused necroses in a distance from this vessel longer than the critical diffusion distance. Therefore, better vascularized tumors exhibited areas with higher necroses along with bigger vessels. These necrotic areas were formerly viable tumor cells that lost their oxygen and glucose supply and became necrotic. In consequence, this leads to bigger well-vascularized tumors with a smaller fraction of vital tissue. The results in this study support this explanation. The smaller proportional vital tumor volumes in CE-CT were found in the better vascularized tumors.

The smaller, non-co-transplanted tumors showed slower growth rates, did not develop poorly supplied areas, and therefore lacked such necroses. The histological findings in this study supported this explanation and are consistent with the findings from Airley et Mobasheri [48].

[18F]FDG-PET indicates these intra-tumor differences by heterogeneous patterns of distribution of [18F]FDG-activity in this study [49]. These patterns may reflect the coexistence of necrosis and vital perfused tumor tissue side by side. Tixier et al. described similar results in their study with a strong correlation between [18F]FDG-PET heterogeneity and blood flow in tumors [50]. Cook et al. found lower overall survival in tumors with highly heterogeneous distribution patterns of [18F]FDG [51]. Co-transplanted tumors in this study exhibited both, higher uptake of glucose in [18F]FDG-PET and better vascularization in histology.

On a molecular level, this constantly new occurring hypoxia in growing tumors may lead to the activation of hypoxia associated genes, as HIF-1 [12, 52]. HIF-1, as described, triggers both angiogenesis [53] and amplified glycolysis [12, 52, 54]. This may result in higher SUVs in [18F]FDG-PET fast growing tumors.

*First explanation in keywords*: better blood supply ➔ faster growth ➔ necrotic and hypoxic regions ➔ activation of angiogenesis and glycolysis.

#### Second explanation

The glucose metabolism of cancer cells is not saturated because of insufficient cancer vascularization. Improvement of this vascularization by an induced angiogenic switch improves both blood and glucose supply. Consequently, this leads to a higher influx and turnover of glucose, because GLUT-1 and HK-II were not saturated before. The correlations between contrast enhancement in vital tumor parts in CE-CT and maximal and mean SUV in tumors support this.

### Imaging vascularization with [18F]FDG and CE-CT in this study

Maybe the regulation of both angiogenesis and glucose metabolism is a result of different pathways. However, [18F]FDG uptake seems to be an indicator for vascularization of malignant tumors. This study could successfully show correlations between glucose metabolism and vascularization. The results are consistent with similar studies [55–57]. Other groups find contrary results for comparison of perfusion imaging and [18F]FDG-PET [58, 59]. Sauter et al. [60] found negative correlations for SUV and MVD but positive correlations for perfusion CT and MVD. Accordingly, imaging parameters of [18F]FDG-PET may be directly connected to the degree of vascularization of tumors. The correlations found between SUV and beads/FOV and SUV and CE in CT in vital tumor support this assumption. Xing et al. [55], Yokobori et al. [61], and Kaira et al. [57] could also show correlations between SUV and MVD in pulmonary tumors. Whereas the latter found only weak correlations in patients with secondary pulmonary tumors. Tateishi et al. [56] also found correlation between SUV in [18F]FDG-PET and MVD in pulmonary tumors. Interestingly, this was not true for benign lesions. Using only two tumor cell lines as in this study avoided the influence of cell characteristics on imaging. All these results may be allegeable if the regulation of both angiogenesis and glucose metabolism is discussed. As described before, both processes are influenced by the same regulator proteins, among others HIF and p53. Based on mutation or hypoxia, HIF in cells becomes stabilized. Both, angiogenesis via VEGF and glycolysis via induction of HKII, GLUT-1, GLUT-3, and GLUT-4 are promoted [12, 16, 34]. The mutation of the P53 gene, often found in cancer cells, rules both processes similarly. The absence of functional p53 (due to mutation) leads to both a stop of inhibition of glycolytic enzymes and to the promotion of angiogenesis [16, 34, 44–46].

In tumors, vascularization is associated with higher aggressiveness. If high [18F]FDG-uptake is associated with higher vascularization, one perhaps can hypothesize with reservations that high [18F]FDG-uptake in some tumors may hint for higher aggressiveness. It was shown recently that local relapses of NSCLC tumors after radiation therapy are significantly more often found in pretherapeutic high [18F]FDG uptake tumor areas [62]. This raises the question which cancer cell traits in these areas lead to both the high glucose metabolism and the high malignant potential. In other tumors, the data for [18F]FDG-uptake and aggressiveness are contrary. Due to different imaging protocols, the measured SUV values are hard to compare. That makes it difficult to establish cut-off values for estimating tumor aggressiveness and prognosis.

### Thresholds in PET imaging

The threshold for defining “metabolic active” is from tremendous meaning at this point. Above described results hold true for a lower threshold of 50% of the particular rat’s brain SUVmean. By using a threshold of 50% of the rat’s tumor SUVmax, both volumes change if tumors contain large parts with low glucose turnover. In non-co-transplanted tumors, this threshold is closer to tumor SUVmean than in co-transplanted tumors. In consequence, nearly the entire tumor volume is defined as “metabolic active.” The significant differences remain between both groups, but in the opposite direction (Table 2). This fact mirrors the finding of heterogeneous [18F]FDG pattern and coexistence of necroses and vital tumor tissue in co-transplanted tumors. The findings in this study also underscore the tremendous meaning of the level of thresholds selected. Their importance increases if they lay the foundation for determination of target volumes for radiation therapy.

### Limitations of this study

Although in this study correlations between imaging parameters in [18F]FDG-PET/CT and vascularization were detected, it must be emphasized that correlations not necessarily mean causality. Especially in [18F]FDG-PET, the abovementioned factors influencing the tracer uptake must be considered. In the high complexity of cancer, conclusions should never be driven on the basis of one single method. In this way, [18F]FDG-PET/CT should be used when estimating vascularization in tumors, as a further piece for a very complex puzzle. A limitation of this study is due to the fact that human tumor xenografts were grown in animals. It changed the original microenvironment of the tumors, but it enabled an easier translation into clinic using human cancer cell lines, the exclusively use of clinically approved scanners and imaging protocols, and by alteration of microenvironment within the same tumor cell line. Although the technique for the estimation of the MVD is well established, it is limited because it only focuses on some hot spots of the tumors and may not represent the entire tumors’ vascularization. The random selection of evaluated tumor areas should have minimized this limitation. The analyzed tumor heterogeneity may not only help to characterize tumors, it may also influence other estimated parameters in an unknown manner. Furthermore, the partial volume effect in PET and CT analyses may hamper some results. Because it is clinically well established, this study only focuses on static SUV estimation. However, dynamic [18F]FDG-PET registration may add further information in subsequent studies. Already dual time point imaging can expand the information obtainable from [18F]FDG-PET imaging. Nakajima et al. were able to differentiate histological types of renal cell carcinomas by early imaging after [18F]FDG application and raise hints for aggressiveness by comparison of [18F]FDG accumulation over time [63]. Wu et al. could show that dual time point analyses in [18F]FDG-PET can be used for further evaluation of bone lesions [64]. Estimating PET parameters from tracer kinetic modeling uses dynamic PET for continuously collecting data during a certain timeline. In order to calculate parameters from the compartment model, an arterial input function is mandatory for calculating tracer kinetics. To avoid the gold standard of blood sampling, most studies used image derived input functions. [18F]FDG is irreversible trapped intracellular. This means [18F]FDG exhibits an unidirectional transfer from the blood into the cells; it is for this reason why for calculations of transfer constants the Patlak analysis can be used [65]. Compared to static or dual time point imaging, the dynamic [18F]FDG-PET much more deepen the information extracted from imaging by determining parameters from tracer kinetic [66]. They can help to further understand pathophysiological mechanisms or the dignity of tumors [66]. Although considering the complexity of the analysis, Wu et al. emphasize that dynamic [18F]FDG-PET is more helpful in discriminating between the dignity of bone lesions [64].

While morphologic imaging exhibits high reproducibility, the results of functional imaging methods like [18F]FDG-PET and perfusion imaging are dependent from the methods and deepness of the selected type of anesthesia. In a preliminary study, the authors evaluated the used imaging protocols that revealed a reliable distribution of [18F]FDG-PET through the whole animal. Several studies compared different application forms of [18F]FDG-PET (e.g., intravenous, i.v. and intraperitoneal, i.p.) and explored factors influencing tracer distribution in rodents [67, 68] and revealed a comparable increase in [18F]FDG-PET uptake in xenograft tumors after i.v. as well as i.p. application [69]. Furthermore, the type of anesthesia influences imaging results. The authors in this study used i.p. ketamine and xylazine after tracer application to minimize the effects on tracer metabolism. Ketamine is not supposed to suppress cardiac output significantly [25] and inotropic and chronotropic effects are dose dependent [70]. Other studies showed that different narcotics may cause worsening of cardiac function [70–72]. Summarized, all anesthetics may cause worsening of cardiac function influence haemodynamics, all with their inherent advantages and disadvantages. Due to the used doses in this study, the authors believe, in line with the literature, that there were no relevant cardiodepressive effects that could have disturbed the distribution of [18F]FDG-PET in this study.

## Conclusions

The results showed the connection between the higher glucose metabolism in tumor areas and the higher angiogenic potential in these tumors. [18F]FDG-PET/CT and CE-CT can be utilized estimating parameters that indicate high tumor vascularization. This study successfully estimated parameters which seems to be connected to tumor vascularization. Linear correlations were not found between all reviewed modalities implicating that imaging modalities should not be replaced by each other. [18F]FDG-PET/CT, CE-CT, and histology detected parameters which seems to be connected to tumor vascularization. Unfortunately, it remains unclear to which molecular basis they refer. Further research concerning the molecular basis of tumor vascularization and its imaging is necessary to optimize diagnostic imaging in order to individualize patient therapy.

## Abbreviations

[18F]FDG-PET/CT: F-18-fluorodeoxyglucose positron emission tomography / computed tomography
CD-31: Cluster of differentiation 31
CE-CT: Contrast-enhanced computed tomography
CM: Contrast agent
DMEM: Dulbecco’s modified Eagle medium
FMISO: Fluoromisonidazole
FOV: Field of view
GLUT: Glucose transporter
HE: Hematoxylin-eosin
HIF: Hypoxia-inducible factor
HK: Hexokinase
HX4: Hydrophilic flortanidazole
i.p.: Intraperitoneal
i.v.: Intravenous
MVD: Microvessel density
NSCLC: Non-small-cell lung carcinoma
RGE: Rat glomerular endothelial
rHU FGF-b: Recombinant human fibroblast growth factor b
rHU VEGF-165: Recombinant human vascular endothelial growth factor 165
SUV: Standardized uptake value
TCA: Tricarboxylic acid cycle
